# Geometric modelling of 3D pore space using curve skeleton: Application to computational microbiology of soil organic matter mineralization

**DOI:** 10.1371/journal.pone.0331031

**Published:** 2025-11-07

**Authors:** Zakaria Belghali, Olivier Monga, Mouad Klai, El Hassan Abdelwahed, Lucie Druoton, Valérie Pot, Philippe C. Baveye

**Affiliations:** 1 IRD, UMMISCO, Unité de Modélisation Mathématique et Informatique des Systèmes Complexes, Bondy, France; 2 Faculty of Sciences Semlalia, Department of Mathematics, Cadi Ayyad University, Marrakech; 3 Laboratoire LIB, Université de Bourgogne Europe, Avenue Alain Savary, Dijon; 4 UMR 1402, ECOSYS, INRAe, Saclay, France; 5 Saint Loup Research Institute, 7 rue des chênes, La Grande Romelière, Saint Loup Lamairé, France; UCAM: Universidad Catolica San Antonio de Murcia, CHINA

## Abstract

Recent advances in 3D X-ray Computed Tomography (CT) sensors have stimulated research efforts to unveil the extremely complex micro-scale processes that control the activity of soil microorganisms. Classical methods for the numerical simulation of biological dynamics using meshes of voxels, such as the Lattice Boltzmann Method (LBM), tend to require long computation times. The use of more compact geometrical representations of the pore space can drastically decrease the computational cost of simulations. Recent research has introduced basic analytic volume primitives to define piece-wise approximations of the pore space to simulate drainage, diffusion, and microbial mineralization of organic matter in soils. Such approaches work well but a drawback is that they give rise to significant approximation errors caused by imposing a priori shapes to represent the pores. In the present article, another alternative is proposed, where pore space is described by means of geometrically relevant connected subsets of voxels (regions) regrouped on the basis of the curve skeleton (3D medial axis). The curve skeleton has been adopted to characterize 3D shapes in various fields (e.g., medical imaging, material sciences, etc.). The few publications that have used it in the context of soils have dealt exclusively with the determination of pore throats. This technique is used mostly to describe shape and not to partition it into connected subsets like in the present work. Here, the pore space is partitioned by using the branches of the curve skeleton, then an Attributed Relational Graph (ARG) is created in order to simulate numerically the microbial mineralization of organic matter, including the diffusion of by-products. Each node of the ARG is attached to an element of the partition (pore) and each arc to an adjacency relationship between pores (connectivity). The graph is valuated in the sense that the attributes related both to geometry and dynamic are linked to nodes and arcs. This new representation can be used for graph-based simulations, which are different from voxel-based simulations.

## 1 Introduction

Soils worldwide contain a very large stock of organic matter, whose mineralization by micro-organisms has the potential to have a sizeable impact, positive or negative as the case may be, on the amount of greenhouse gases released to the atmosphere, and therefore on climate change [1,2]. Over the last four decades, the importance of soils in that context has prompted a very significant research effort on the description and prediction of the fate of organic matter in subsurface environments. Initially, the modelling component of that effort focused on the development of black-box models like Century or RothC, in which the metabolic activity of microorganisms was described simply via first-order kinetic equations, without a detailed accounting of the growth and spatial distribution of the biomass [3,4]. After the turn of the century, it became clear that much more attention needed to be paid to where in the soil pores the micro-organisms were located relative to the organic matter they fed on [5,6]. Fortunately, concomitant technical advances in X-ray computed tomography (CT) enabled researchers to routinely obtain three dimensional gray scale pictures of soils at progressively increasing resolutions, from which, after the development of suitable thresholding algorithms [7–11], binary images could be obtained, providing micro-scale information on the geometry and topology of soil pores in which the microorganisms reside. Over the last decade, this information, complemented by a range of other micro-scale (microscopic and spectroscopic) measurements [6,12], has stimulated extensive research on modeling the soil microbial processes [7–10,13–18].

One of the key hurdles that researchers have faced in the computational microbiology of soil organic matter mineralization is related to the sheer size of the image files that result from CT scans, with numbers of voxels in the dozen of millions (up to hundreds for some data) and upward for the highest attainable resolutions. The Lattice Boltzmann Method (LBM), which is generally the method of choice to describe the movement of water and the diffusion of nutrients in the pore space of soils, is very accurate [10,19] but requires computers with very large memories and is very demanding in CPU time with currently available computer technologies. Some efforts have been devoted toward the development of advanced computer vision and computational geometry tools in order to obtain better, less memory-intensive representations of the pore space [15,16,20–22]. These tools aim to provide compact, intrinsic, and relevant geometrical representations from the original set of voxels, which can lead to a considerable speeding up of the numerical simulation algorithms. Unlike older pore network models, which are based on an idealized pore space representation, the more recent methods take into account the exact geometry of the pore space as revealed by 3D CT images.

An alternative consists of defining an intrinsic analytic piece-wise approximation of the pore space using basic volume primitives. A drawback of most analytic approaches lays in the a priori choice of given geometric primitives. Within a pore network modelling context, a well-known algorithm is the Maximal Ball algorithm [23–25]. It consists of identifying the maximal spheres contained in the pore space and then approximating the volume by a subset of the spheres. This algorithm has been modified by Dong and Blunt [24], who proposed a faster way to determine the set of maximal balls. In their approach, the network representing the pore space is composed of balls and cylinders associated with pores and throats. The biological simulation is then performed using this network. Another scheme alternative is to approximate the pore space with an optimal subset of maximal spheres and cylinders [10,14,16,18,20,26]. This optimal set is derived from the minimum set of balls (in a cardinal sense) recovering the λ-skeleton. Afterward, a numerical simulation of microbial mineralization is performed using the ball network [14,17,27].

Recently, several authors have proposed using more sophisticated geometrical primitives such as ellipsoids and generalized cylinders. For instance, the algorithm described by Kemgue and Monga [15,28] includes two steps. First, maximal spheres are clustered using the k-means partitioning method. Afterward, each cluster is approximated with a primitive. Finally, an optimal region-growing stage allows one to reduce the number of primitives. Regarding air-water interface extraction, a pore space representation with spheres or ellipsoids gives good results. Within other application contexts, including in medicine, several publications also deal with complex volume shape modelling using sophisticated primitives like ellipsoids [12,13,22,29–34]. Unfortunately, the experience shows that these approaches cannot easily be extended to the pore space geometrical modelling in soils due to the higher shape complexity in these systems.

Nevertheless, a drawback of the approximation methods using geometric primitives is that, to be computationally advantageous relative to the LBM, they provide only a somewhat crude representation of the convoluted geometry of the pore space, which cannot be fully represented due to the comparative simplicity and compactness of spheres, for example. One of the main problems is the loss of voxels at the edges of the pore space. Practically, this loss of voxels lies in the range 5% to 20% of the pore space depending on the data (see [15]).

An alternative to using geometrical primitives consists of partitioning the soil pore space into connected subsets of voxels computed on the basis of the curve skeleton. In two dimensions, the so-called medial axis (2D curve skeleton) of a shape is defined as the locus of the centers of maximal inscribed disks. By definition, a disk is maximal if it is not strictly included in another disk inside the shape. In three dimensions, things are a little more complicated. The medial surface (3D surface skeleton) corresponds to the center of the maximal inscribed balls.. This medial surface is often referred to as the surface skeleton and the process of obtaining it is termed the “skeletonization” of a geometrical shape [35,36]. These different concepts (2D and 3D medial axis) have been widely used in computational geometry since Blum’s landmark paper [35], and one can find in the literature various ways to compute the surface skeleton, depending on the initial description of the shape: the triangular mesh, subset of voxels in a 3D image, point cloud sampling the boundary of the shape and so on. For instance, regarding shapes represented with a set of voxels in a 3D image, Xia and Tucker [37] computed a distance map to the shape boundary by solving the Eikonal equation with the Fast-Marching method. This distance map gives, for each voxel in the shape, the geodesic distance (i.e., the length of the shortest path inside the shape) to the boundary of the shape. The voxels of the surface skeleton are then extracted with the Laplacian of this map. Melkemi [38] and Amenta et al. [39] describe the Power Crust algorithm, which approaches the medial axis of a point cloud sampling the shape boundary. The surface skeleton can also be approximated using the 3D Delaunay Triangulation of a finite set of points forming a discretization (sampling) of the shape boundary. The set of the centers of the “Delaunay” spheres circumscribed to the Delaunay tetrahedra, and included within the shape, gives an approximation of the skeleton [16,20,40].

The sensitivity of the surface skeleton to little changes in the shape boundary is a drawback for several applications. There has been a tendency in the literature to invoke more robust, less sensitive approaches than the surface skeleton to address various tasks such as animation, motion tracking, shape recognition and analysis. When the details of the selected shape are irrelevant, the surface skeleton is simplified using the hierarchical removal of small maximal spheres. Such strategies are related to the notion of λ-skeleton [41], which presents undeniable interest in a number of contexts. However, in the type of situation that concerns us, small maximal spheres can be filled with water, and thus diffusion processes can take place in such pores. Therefore, one should exercise caution when simplifying the computation of the surface skeleton in porous media and soils in particular.

Although the surface skeleton provides a sound foundation for skeletonization efforts, experience has shown that another type of skeleton, the curve skeleton, which is a 1D manifold rather than a 2D one, is much easier to take into account for modeling purposes. This is the main reason why the curve skeleton has been widely used for the representation of geometrical shape [42–44]. Several definitions have been proposed for this second type of skeleton, which is alternatively referred to as a “curve” or “curvilinear” skeleton in the literature. It can be defined as a 1-dimensional subset of the surface skeleton, fulfilling a supplementary condition, like the singularity of the Hessian of the distance map. There are other definitions based on morphological operators, like erosion and homotopic thinning. Several discrete or continuous curve skeleton extraction methods have been proposed in the fields of discrete geometry and shape analysis. To this end, [24] describes a “homotopic thinning” algorithm. Zwettler et al. [45] adapted this algorithm in the specific case of tubular surfaces representing blood vessels for medical diagnostics. Sobiecki et al. [46,47] compared the different methods for computing curve skeletons. They give a comparison of voxel-based methods with mesh contraction-based methods. Both are controlled by geometrical criteria such as the homotopy, the thickness, and the preservation of details. The use of the curve skeleton to segment 3D shapes is easy to illustrate and implement, but it has been explored only recently. Reniers and Telea [44,48] segmented the shape according to the critical points of the curve skeleton. The critical points were defined as triple cross road points, i.e., belonging to at least three branches of the curve skeleton. The curve skeleton is then segmented using the geodesic distances (length of the shortest paths inside the volume) of the critical points to the boundaries. Following the same principle, Brunner and Brunnett [26] presented a mesh partitioning algorithm combining the voxelization and the homotopic thinning. On that basis, they considered shapes for which the curve skeleton is relatively simple. However, that is generally not the case in soils.

Indeed, in the earth sciences, Lindquist and Venkatarangan [49] use the medial axis (3D curve skeleton) in order to analyze the spatial distribution of pores in geological materials. Using this approach, Youssef et al. [21] address the quantitative 3D characterization of the pore space of real rocks within the context of petroleum extraction. The authors compare the results of the measurements with those of simulations for various porous media like sands and carbonates. Their work illustrates the nice geometric properties of the curve-skeleton to model complex shapes within other contexts than soil science.

Then, the curve skeleton based representation of the pore space ensures the inclusion of all voxels present in the pore space [35,42,43], and can be used to model the microbial mineralization of organic matter in soils. Earlier work [14] uses also attributed relational graphs to represent pore space but the nature of the graph is different in this study. Subsequently, following the methodology described in [18], we compare the modelling results obtained via this new approach with those produced by the classical Lattice-Boltzmann method. The difference of the present research with the one of [17] lies in the geometric representation of the pore space, which is quite different. In [17] the pore space was described thanks to an optimal ball network approximating pore space. In the present work, we represent it with a partition derived from the curve skeleton. We address implicitly the issue of connectivity by using the adjacency graph of the curve skeleton based regions. These regions define a complete partition of the pore space and therefore by definition no pore space connectivity is lost. This new representation is more compact and also more precise because, by design, it does not involve approximation errors. Its increased compacity allows to decrease computing time by a ratio of ten to twenty (depending on the data).

In this general context, the objective of the present article is to describe the partitioning of the soil pore space into connected subsets of voxels computed on the basis of the curve skeleton. We outline the computation of the curve skeleton, the partitioning of the curve skeleton into simple branches, the building of the graph representing the pore space, the method used to compute the overall conductance of a soil sample, and how we validate the choice of a time step for an implicit numerical scheme using an explicit numerical scheme. The region-based model is then used to simulate the process of microbial mineralization in soils, and is compared to the predictions of models based on geometric primitives. Lastly, we discuss future avenues for research.

## 2 Methods and approaches

### 2.1 Summary

**Fig 1:**
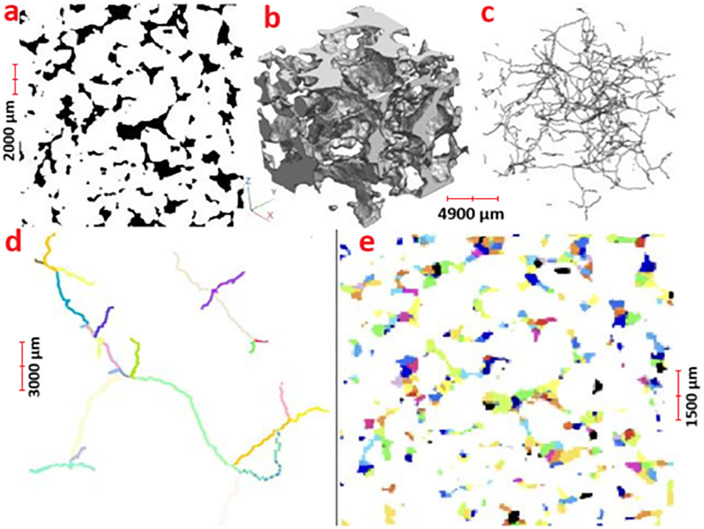
Top left: 2D cross-section of the sandy loam soil (dataset 1, voxels; where the voxels associated with the pore space are colored in black (of the whole image). Top middle: perspective view of a small part of the whole pore space. Top right: perspective view of the corresponding curve skeleton. Bottom left: segmentation of the curvilinear skeleton into simple branches; zoom on some simple branches (18508 simple branches in total). Bottom right: cross section of the partitioning of the pore space based on the curve skeleton where each color is attached to a region.

**Fig 2:**
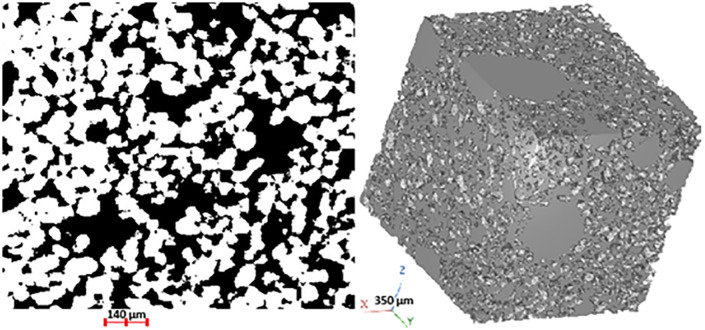
Left: perspective view of the pore space. Right: 2D cross section of Fontainebleau sand (**dataset 3**, low porosity, 512×512×512; 4.5μ𝐦×4.5μ𝐦×4.5μ𝐦); pore space voxels are colored in black (42% of the whole image).

In the present work, we used both binary and gray images. This segmentation was computed and facilitated by the use of classical image processing algorithms (Median filtering, Otsu method…) [34]. Typically, the pore space voxel-based representation encompasses dozen and even hundreds millions of voxels (see Figs 1a, 1b, 2, S1 Fig).

**Fig 3:**
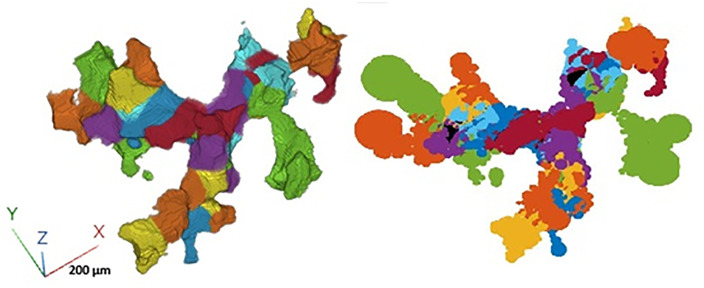
Fitting of the ball and curve skeleton models; left figure (a): A zoom-in of the 3D projection of a portion of the partitioned image depicted in Fig 1. The 3D projection shows better than the 2D images (Fig 1) how the partitioned pore regions are connected. Each color corresponds to a region attached to a simple branch of the curve skeleton.; right figure (b): balls labeled with the corresponding region.

**Fig 4:**
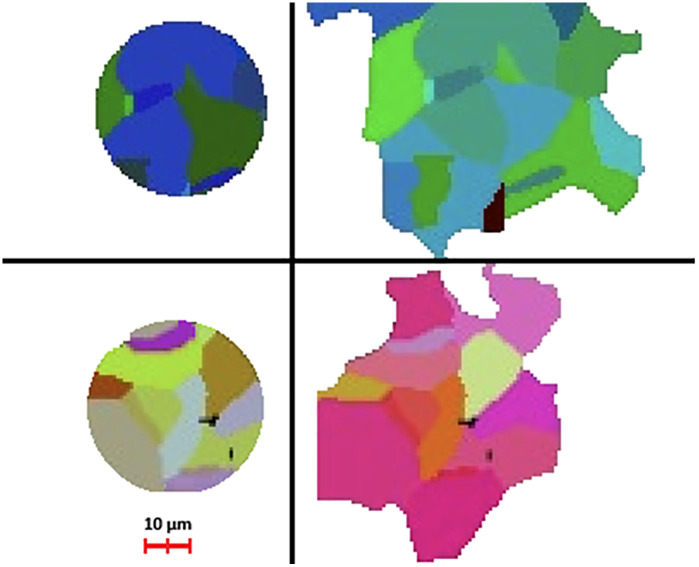
Illustration of the two biggest balls where the micro-organisms are located (dataset 3) for the microbial decomposition simulation (2D cross sections); up left: the biggest ball colored by the parts of the regions which intersect it; up right: the (entire) regions intersecting the biggest ball; bottom left: the second biggest ball colored by the parts of the regions which intersect it; bottom right: the regions intersecting the second biggest ball. Round 85% of the ten biggest regions are included within the two biggest balls. https://free3d.com/.

**Fig 5:**
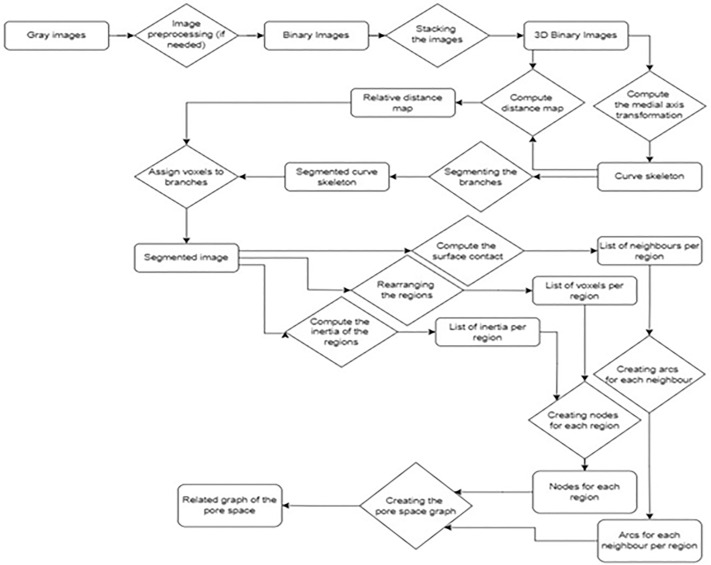
Schematic representation of the main steps from gray images to the corresponding graph of the segmented regions.

**Fig 6:**
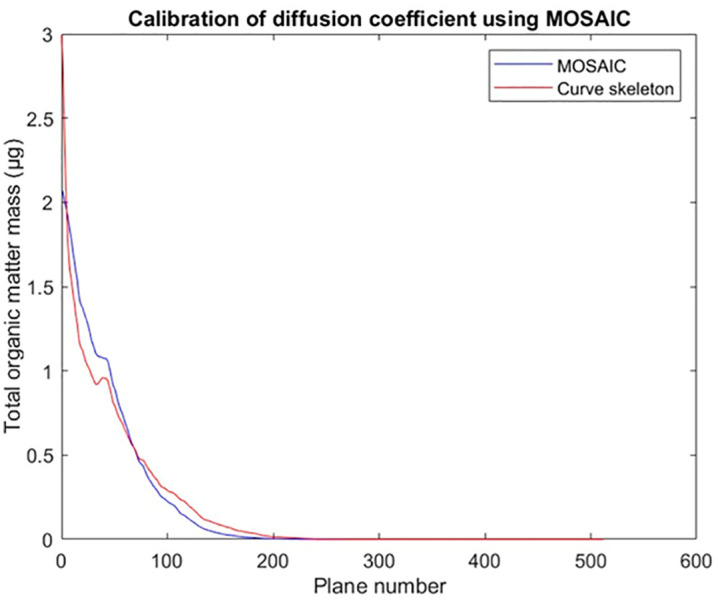
Simulation results for diffusion only (data set 1) used to calibrate the overall conductance to be used in later simulations. The X-axis displays the number of planes within the image (512 planes in total), whereas the Y-axis displays the total mass of organic matter within each plane. At the start, 100 µg of carbon were introduced within the first two planes. The total simulation time was 1.783 hours. The optimal value of the diffusive overall conductance coefficient was determined to be equal to 0.35, with an intercorrelation of 0.9818. We notice that after 1.783 hours only the 300 first planes were reached.

**Fig 7:**
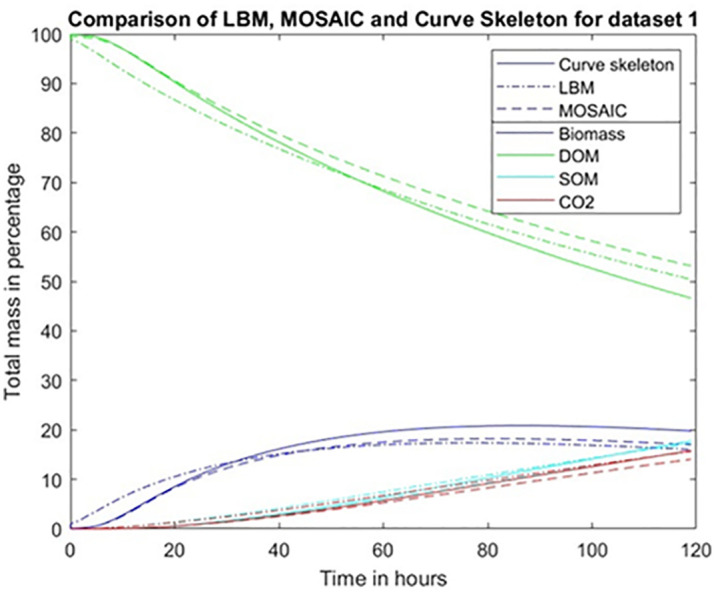
Comparison of different simulations of the microbial mineralization for dataset 1 over a total simulation time of 5 days. The Y axis displays the mass of several components of the system expressed as a percentage of the total initial carbon mass. Simulations were carried out in the “curve skeleton” method with a diffusive conductance coefficient equal to 0.35.

**Fig 8:**
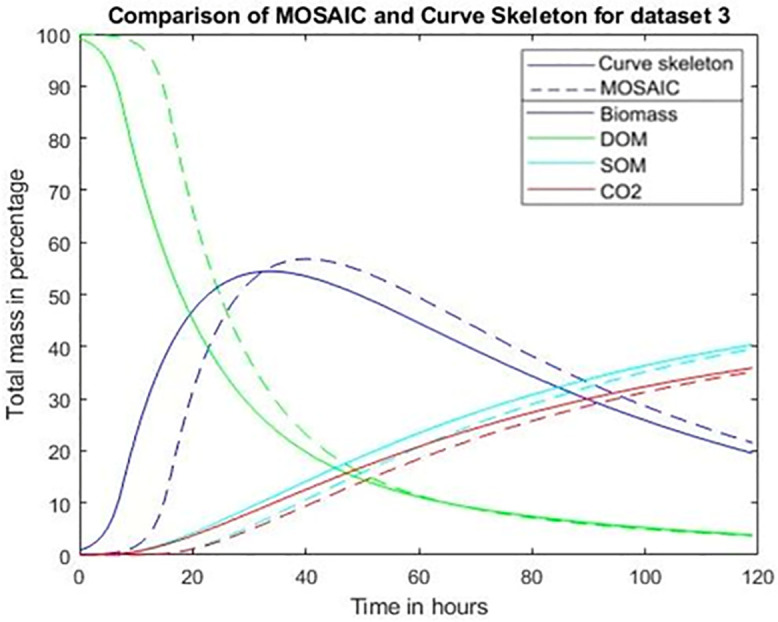
Comparison of the simulations of microbial mineralization for dataset 3 (low sand porosity) using the ball based MOSAIC program and the novel approach introduced here, based on the partitioning of the pore voxels on the basis of the curve skeleton. The simulations extended over 5 days and were carried out in the “curve skeleton” method with a diffusive conductance coefficient equal to 0.35 for the curve skeleton method and 0.6 for the balls method. The initial masses (micro-organisms, dissolved organic matter) and also the diffusion coefficient were adjusted according to the image resolution. X-axis and Y-axis represents respectively time expressed in hours and the masses expressed in percentage of the total initial masses. Solid line curves and dotted line curves correspond respectively to the curve skeleton model and to the balls model. Dark blue curves, green curves, red curves, light blue curves correspond respectively to microorganisms, dissolved organic matter (DOM), carbon dioxide (CO^2^), slow organic matter (SOM). Same as for dataset 1 the microbial degradation model take into account DOM and SOM but not FOM.

We deal with the geometrical modelling of pore space from voxels based description and its application to biological dynamics simulation. In the first step, we compute the curve skeleton of this set of voxels [50] (see Fig 1c). In the second step, we segment the curve skeleton into the maximal simple branches (see Fig 1d). In the third step, we identify for each voxel the simple branch that is the closest in an Euclidean distance sense. Afterward, we attach to each simple branch the corresponding set of voxels. In the fourth step, we label (i.e., partition) the pore space into connected sets of voxels (regions) (see Figs 1e, 3, 4, S2, S4 Fig). In the fifth step, we build an attributed adjacency graph of regions from the partition where each node is attached to a set of connected voxels corresponding to a pore (see Fig 5). Finally, we use this pore space representation to carry out the numerical simulation of microbial mineralization, as shown in Figs 6, 7, 8, S3, S5 Fig. In section 2.2 we describe the geometric modelling method of the pore space by means of the curve skeleton. Section 2.3 presents the framework for using the geometric modelling to simulate microbial decomposition.

### 2.2 Geometrical modelling of the pore space using curve skeleton

#### 2.2.1 Computation of the curve skeleton.

Let S be the set of voxels forming the pore space. Let B be the surface border of S. We assume without loss of generality that S is connected. If it is not the case, we work separately on each connected component of S. We compute the curve skeleton of S by means of successive erosions using the homotopic thinning algorithm [51].

We define the distance transform DT_S_ for a three dimensional set of voxels S∊ R^3^ with boundary points B:


$$\text{DTs}(\text{x}\in \text{S})={\text{min}}_{y\in \text{B}}\;|\;|\;\text{x}-\text{y}\;|\;|$$ (1)

Next, in mathematical terms, the definition of the curve skeleton, known as Medial axis MA_S_, is:


MAs ={x∈S | ∃ (f1,f2,f3)∈B3,f1≠f2≠f3; // f1 −x// =// f2 −x//= // f3−x//=DTs(x)}  (2)

As mentioned in [21,50,51], at each stage of the erosion process, voxel (i,j,k) is deleted if the following conditions hold:

- (i,j,k) lies on B (S boundary), i.e., at least one of its 26 neighbors lies outside S.
- (i,j,k) is not an ending point (an ending point has only one neighbor), regarding the 26-neighborhood.
- (i,j,k) removal does not modify the Euler characteristics (topological condition) [52].
- (i,j,k) removal does not modify the number of connected components (topological condition).

The Euler characteristic is a topological invariant that is preserved when computing the curve skeleton (see references [25,52]). The skeleton is computed from a volume that has a number of connected components, a number of cavities, and a number of holes. Euler’s characteristic is a topological feature that depends on these three values. When the volume is discretized by a set of voxels, there is an equivalent definition using the number of vertices, edges, faces, and octants, as outlined in [25]. Ensuring that this characteristic remains invariant through the skeletonization algorithm helps control, for example, the prevention of introducing holes into the skeleton or increasing the number of connected components by progressively thinning the volume.

This erosion process (thinning) is iterated until the fixed point is reached (idempotence). The homotopic thinning algorithm is robust and works for any shape represented by voxels, even for disconnected B with connected S (volume shape with holes). By construction, the curve skeleton thickness is one voxel. In general, the curve skeleton of a given shape is unique, but some very symmetric shapes can have several curve skeletons, for instance the cube [0 9]^3^minus the concentric cube [3 6]^3^. For these cases, the homotopic thinning algorithm comes up with a curve skeleton depending on implementation details, like the order in which boundary voxels of S(B) are considered. Such cases are unlikely when modelling natural shapes such as soils.

#### 2.2.2 Extraction of the pores: Partitioning of the curve skeleton into simple branches.

We propose to segment the curve skeleton in order to extract the pore network.

The curve skeleton is represented by a set of voxels connected. Neighboring voxels are linked by a face, an edge, or a point, we use the 26-connectivity.

In the curve skeleton, there are three different kinds of voxels:

- Ending voxels are the nodes with only one neighbor.
- Simple voxels are the nodes with exactly two neighbors.
- Interior voxels are the nodes with more than two neighbors.

A branch is a maximal set of connected simple voxels. Ending voxels and interior voxels are located at the extremity of branches. Interior voxels lie at the interconnection between branches. Fig 1.c shows an example of skeleton representation. We segment it into a set of branches using a straightforward algorithm. Thus, we get a set of branches forming a partition of the curve skeleton.

#### 2.2.3 Building of the graph representing pore space.

Afterward, each voxel of S is associated with the nearest branch of the curve skeleton in the sense of Euclidean distance. The result is a partition of S composed of regions forming a practically connected set of voxels. In very uncommon cases, where the set of voxels attached to a branch would not form a connected component, one can split it into connected sets. Another way of tackling this problem would be to use the geodesic distance instead of the Euclidean one, as proposed in [27,53]. By this way, the pore space can be partitioned into connected sets of voxels corresponding to a single branch of the curve skeleton. The partitioning of S is directly defined by the nearest sets attached to the branches. The graph of regions defining the pore network is built as follows. Each node of the graph is attached to a region (pore) corresponding to a branch of the curve skeleton. Each arc of G is attached to a pair of neighboring regions.

The adjacency relationships are computed by building a 3D image L(i,j,k) where each voxel is attached to the label of the region where it is included. In case of (i,j,k) does not belong to S, L(i,j,k) is set to 0. The arcs of G and the area of the contact surfaces between adjacent regions (pores) are determined by means of a one-pass scanning. For each voxel (i,j,k) of S we look for the three neighbors: (i + 1,j,k), (i,j + 1,k), (i,j,k + 1) and for each neighbor belonging to S, we update the graph adjacencies (arcs) and the areas of the contact surfaces (arc features) by looking to the values of the label image (L). If the two labels are the same, we do nothing. If the two labels are different, we eventually create a new arc in the graph and increment the area of the contact surfaces between the two regions (Fig 5).

This algorithm finds all neighboring relationships between regions and all the corresponding areas of the contact surfaces. It is used because not all edges of G can be created considering only the branches of the curve skeleton. Indeed, some regions may share a common boundary surface although their branches do not have a common interior node. Neglecting regions adjacencies, as was done in [26], could be a significant drawback for many applications. For instance, in the case of diffusion simulations, it would imply that flows would transit only along branches of the curve skeleton.

### 2.3 Simulation framework: Microbial mineralization of the organic matter including diffusion processes from the graph of regions

#### 2.3.1 Principle.

From the geometric modelling stage (see 2.2) we get an adjacency valuated relational graph such that:

- Each node is attached to a connected set of voxels (region, pore) corresponding to a branch of the curve skeleton; we associate to each node the coordinates of the inertia center of the set of voxels and its volume (number of voxels).
- Each arc is attached to an adjacency relationship between two regions (pores); we associate to each arc the area of the contact surface between the two corresponding regions (pores).

Afterward, we use the graph G, representing the pore space, to simulate microbial mineralization. The global scheme of the numerical simulation is the same as the one described in [17]. The principle is to discretize the process in time and to successively implement transformation and diffusion processes. The diffusion processes are implemented by means of the implicit numerical scheme (see 2.3.4). The computational cost is roughly proportional to the number of graph nodes.

As mentioned in the sequel, the explicit scheme is used for validating the time step of the implicit scheme. Indeed, the choice of using an implicit scheme or an explicit scheme depends on the data. Practically, it is better (in terms of computational cost) to use an explicit scheme for very small time steps (less than one second) and better to use an implicit scheme for larger time steps (more than a few seconds). In our experiments, the time step is 30 seconds that makes implicit scheme be much more efficient.

However, the geometrical representation of the pore space is different than in previous approaches. The authors in [17] described the pore space by the minimal set of balls covering the surface skeleton. The drawback of that approach is that a part of the pore space (typically 15%) is lost due to the piecewise approximation errors. The geometrical representation adopted in the present article covers completely the set of voxels corresponding to the pore space. In particular, we get the exact values for the contact surface areas between two pores.

Insofar as microbial growth is concerned, we apply the model described in [18]. We split the diffusion process from the transformation (reaction) process and we implement sequentially the two processes. The basic reason is that a parallel implementation would imply the use of too small time steps that would increase considerably the computational cost. Moreover, only the diffusion process can be implemented using a backward Euler numerical scheme (implicit numerical scheme). Practically, we use the same time step (enough small, around 30 seconds) for diffusion and transformation (reaction) processes as in [8,10,14,17].

#### 2.3.2 Model equations.

The Partial Differential Equations (PDE) model equation are as follow:


$$\;\left\{ \;\;\;\begin{array}{c} \frac{dx_{i}^{\text{1}}(t)}{dt}\;=-\rho .x_{i}^{\text{1}}(t)-\mu .x_{i}^{\text{1}}(t)\\ +\frac{v_{DOM}\;{.x}_{i}^{\text{2}}(t)}{K_{DOM}\;+x_{i}^{\text{2}}(t)}.x_{i}^{\text{1}}(t)\\ \begin{array}{c} \frac{dx_{i}^{\text{2}}(t)}{dt}\;=D_{\text{c}}.\Delta (x_{i}^{\text{2}}(\text{t}))+\;\beta .\mu {\;.x}_{i}^{\text{1}}(t)-\frac{v_{DOM}.x_{i}^{\text{2}}(t)}{K_{DOM}\;+\;x_{i}^{\text{2}}(t)}.x_{i}^{\text{1}}(t)\\ +\;v_{SOM}{.x}_{i}^{\text{3}}(t)+v_{FOM}{.x}_{i}^{\text{4}}(t)\\ \frac{dx_{i}^{\text{3}}(t)}{dt}\;=(\text{1}\;-\;\beta )\mu \;{.x}_{i}^{\text{1}}(t)\;-\;v_{SOM}{.x}_{i}^{\text{3}}(t) \end{array}\\ \frac{dx_{i}^{\text{4}}(t)}{dt}\;={-v}_{FOM}.x_{i}^{\text{4}}(t)\\ \frac{dx_{i}^{\text{5}}(t)}{dt}\;=\;\rho .x_{i}^{\text{1}}(t) \end{array}\right.\;$$ (3)

where ${\;x}_{i}^{\text{1}}(t)$, $x_{i}^{\text{2}}(t)$, $x_{i}^{\text{3}}(t)$, $x_{i}^{\text{4}}(t)$, $x_{i}^{\text{5}}(t)$ are respectively the masses of MB (Microbial Biomass), DOM (Dissolved Organic Matter), SOM (Solid Organic Matter), FOM (Fresh Organic Matter), CO_2_ (Carbon Dioxide) for the node i of the graph at time t (within the next subsection: ${\;x}_{i}^{j}(t)=b_{j}$); ρ is the; μ is the mortality rate; $v_{DOM}$ and $K_{DOM}$ are respectively the maximum growth rate of MB and the constant of half saturation of DOM; $D_{c}$ is the diffusion coefficient of DOM in water; β is the proportion of MB returning to DOM (the other fraction (1−β) returns to SOM); $v_{FOM}\;$and $v_{SOM}$ the hydrolysis rate of FOM and SOM; $\Delta (x_{i}^{\text{2}}(t))$ the Laplacian of the DOM (diffusion term). We notice that in the results section, the initial FOM and SOM masses were set to 0 (no FOM).

In our implementation we use a splitting scheme for transport and reaction terms and different technical solutions for the subproblems.

#### 2.3.3 Transformation process (microbial decomposition) discretization.

Let us define the set of biological parameters at a graph node $\left(b_{\text{1}},b_{\text{2}},b_{\text{3}},b_{\text{4}},b_{\text{5}}\right)$ where: $b_{\text{1}}$ is the microbial biomass, $b_{\text{2}}$ is the mass of dissolved organic matter (DOM), $b_{\text{3}}$ is the mass of soil organic matter (SOM), $b_{\text{4}}$ is the mass of fresh organic matter (FOM), $b_{\text{5}}$ is the mass of carbon dioxide. If v is the volume of the primitive attached to the node then c= $\frac{b_{\text{2}}}{v}$ is the concentration of DOM within the region attached to the node. DOM comes from the decomposition of SOM (slow decomposition) and of the FOM (fast decomposition). The microorganisms grow by assimilating DOM, and end up producing carbon dioxide. Afterward, a part of the biomass is transformed into SOM and DOM (mortality process).

For each region attached to a graph node, we get:


$$\begin{array}{c} \left\{ \;\;\;\;\begin{array}{c} b\text{1}(t+\delta t)=\;b\text{1}(t)-\;\rho b\text{1}(t)\;\delta t-\;\mu \;b\text{1}(t)\;\delta t+\;\frac{vDOM\;\;c}{\kappa b+\;c}\;b\text{1}(t)\;\delta t\\ \;b\text{2}(t+\delta t)=\;b\text{2}(t)+\;\rho m\;\mu \;b\text{1}(t)\;\delta t-\;\frac{vDOM\;\;c}{\kappa b+\;c}\;b\text{1}(t)\;\delta t+\;vSOM\;b\text{3}(t)\;\delta t+\;vFOM\;b\text{4}(t)\;\delta t\\ b\text{3}(t+\delta t)=\;b\text{3}(t)+\left(\text{1}-\;\rho m\right)\;\mu \;b\text{1}(t)\;\delta t-\;vSOM\;b\text{3}(t)\;\delta t\\ b\text{4}(t+\delta t)=\;b\text{4}(t)\;-\;vFOM\;b\text{4}(t)\;\delta t\\ b\text{5}(t+\delta t)=\;b\text{5}(t)\;+\;\rho \;b\text{1}(t)\;\delta t \end{array}\right.\;\;\;\;\;\;\; \end{array}$$ (4)

where ρ is the respiration rate, μ the mortality rate, $\rho_{m}$ the proportion of MB returning to DOM (the other fraction returns to SOM), $v_{FOM}$and $v_{SOM}$the decomposition rate of FOM and SOM, $v_{DOM}$and $\kappa_{b}$ respectively the maximum growth rate of MB and constant of half saturation of DOM.

In the simulations, following [17,34], we do not consider the FOM pool (the FOM mass is set to 0) but only DOM and SOM (SOM mass is set initially to 0).

#### 2.3.4 Diffusion process discretization: Euler forward (explicit) and backward (implicit).

The diffusion processes are implemented by means of Euler backward (implicit) or forward (explicit) schemes.

The implicit numerical scheme is formulated as follows. We note: $\Theta_{ij}=D_{c}\frac{s_{ij}}{d_{ij}}\delta t$ where $D_{c}$,$s_{ij}$, $d_{ij}$,δt are respectively the diffusion coefficient, the area of the contact surface between node (region, pore) i and j, the distance between the two inertia centers of regions i and j, and δt the discretization time step.

If we denote by $c_{i}$, $v_{i}$,$N_{i},$ $\vartheta \left(N_{i}\right)$, respectively, the concentration at node i, the volume of the node i, the node i and the neighbor of the node $N_{i}$, the relationship between concentrations at successive iterations can be expressed as follows:


$${\left[\begin{array}{c} c_{\text{1}}\\ c_{\text{2}}\\ \vdots \\ c_{p} \end{array}\right]}^{k}=(\begin{array}{ccc} \frac{\text{1}}{v_{\text{1}}} & \cdots & \text{0}\\ \vdots & \ddots & \vdots \\ \text{0} & \cdots & \frac{\text{1}}{v_{p}} \end{array})(\begin{array}{ccc} v_{\text{1}}+\sum_{N_{j}\in \vartheta \left(N_{\text{1}}\right)}\Theta_{\text{1},j} & \cdots & -\Theta_{\text{1},p}\\ \vdots & \ddots & \vdots \\ -\Theta_{p,\text{1}} & \cdots & v_{p}+\sum_{N_{j}\in \vartheta \left(N_{p}\right)}\Theta_{p,j} \end{array}){\left[\begin{array}{c} c_{\text{1}}\\ c_{\text{2}}\\ \vdots \\ c_{p} \end{array}\right]}^{k+\text{1}}$$ (5)

where $c_{i}^{k}=\;\left(\text{1}+\frac{\text{1}}{v_{i}}\sum_{N_{j}\in \;\vartheta (N_{i})}\Theta_{ij}\right)c_{i}^{k+\text{1}}-\frac{\text{1}}{v_{i}}\sum_{N_{j}\in \;\vartheta (N_{i})}\Theta_{ij}c_{j}^{k+\text{1}}$, p is the number of regions (graph nodes), k is the iteration number (t = t_0 +_ kδt; t_0_ is the initial time).

We solve the above system with help of the conjugate gradient method. We can check the pertinence of the time step value for the implicit numerical scheme using the explicit numerical scheme. In a previous paper, we proposed to implement an implicit numerical scheme using directly the graph of Monga et al. [42]. In the present article, we use a matrix representation yielding substantial computational gain. For the explicit numerical scheme, with the same calculation scheme as in section 2.3.2, we get:


$${\left[\begin{array}{c} c_{\text{1}}\\ c_{\text{2}}\\ \vdots \\ c_{p} \end{array}\right]}^{k+\text{1}}=(\begin{array}{ccc} \frac{\text{1}}{v_{\text{1}}} & \cdots & \text{0}\\ \vdots & \ddots & \vdots \\ \text{0} & \cdots & \frac{\text{1}}{v_{p}} \end{array})(\begin{array}{ccc} v_{\text{1}}-\sum_{N_{j}\in \vartheta \left(N_{\text{1}}\right)}\Theta_{\text{1},j} & \cdots & \Theta_{\text{1},p}\\ \vdots & \ddots & \vdots \\ \Theta_{p,\text{1}} & \cdots & v_{p}-\sum_{N_{j}\in \vartheta \left(N_{p}\right)}\Theta_{p,j} \end{array}){\left[\begin{array}{c} c_{\text{1}}\\ c_{\text{2}}\\ \vdots \\ c_{p} \end{array}\right]}^{k}$$ (6)

where $c_{i}^{k+\text{1}}=\;\left(\text{1}-\frac{\text{1}}{v_{i}}\sum_{N_{j}\in \;\vartheta (N_{i})}\Theta_{ij}\right)c_{i}^{k}+\frac{\text{1}}{v_{i}}\sum_{N_{j}\in \;\vartheta (N_{i})}\Theta_{ij}c_{j}^{k}$, p is the number of regions (graph nodes), k is the iteration number (t = t_0 +_ kδt; t_0_ is the initial time).

The drawback of the explicit numerical scheme is that it requires very small time steps to avoid negative values. Thus, we use it only to check the validity of the time step of the implicit numerical scheme.

##### 2.3.5 Diffusive overall conductance computation: by comparison with voxel based scheme

When Fick flows are defined between two regions, we must multiply them with a coefficient $\propto_{i,j}$ called diffusive overall conductance, following [17]. In order to define $\propto_{i,j}$, we calibrate them by comparison with the diffusion simulation using the balls network and LBM. Practically, we find that we can set $\propto_{i,j}$ to a constant value α. Therefore, the equation corresponding to Fick flow becomes:


$$F_{i,j}=\propto_{i,j}\frac{-D_{c}S_{i,j}\Delta c_{ij}\delta t}{d_{i,j}}$$ (7)

The calibration principle is to adjust such a manner that the diffusion using the curve skeleton-based pore network description fits with the ones provided by the Lattice-Boltzmann Model (LBM) and the balls network methods. Practically, we define diffusion benchmarks and optimize the correlation between the outputs. In this way we find out that setting $\propto_{i,j}$ to the constant value 0.35 allows good fitting results.

The most precise way to define the diffusive conductance coefficient would be to compute it for each pair of adjacent regions. The principle would be to run diffusion process using a voxel representation in order to make it fit with regions representation.

We will explore this scheme in a forthcoming paper. However, when the sizes and shapes of the regions are relatively comparable, a mean value of the diffusive conductance coefficient can be used, at least for same geometric model (balls, curve skeleton based regions), without altering too much the precision of the results.

### 2.4 Modelling scenarios

#### 2.4.1 Data.

We use four different data sets (see Table 1) in the simulations and present results for two (datasets 1 and 3). The scenarios included jointly diffusion and degradation, which are simulated using the region graph following [17], based on the ball network. The first dataset, already used by Monga et al. [17,42], consists of an image of a sandy loam soil (sand, silt, clay: 71%, 19% and 10% soil mass, respectively) from the Bullion Field, an experimental site situated at the James Hutton Institute in Invergowrie (Scotland). A detailed description of the soil samples and of the methods used to produce CT images can be found in [51]. Data sets 2 and 3 consist of two CT images of quartz sand obtained in the Fontainebleau forest, corresponding respectively to high and low porosities. The size of these 3D CT images is 512×512×512 and the resolution 4.5μm×4.5μm×4.5μm, and their porosities are 57% and 42% respectively [44]. The fourth dataset is a Carbonado Diamond downloaded from the Digital Rocks portal. It is found only in placer deposits and Mesoproterozoic meta conglomerates in Bahia, Brazil [54].

**Table 1:** Comparative table about the characteristics of the different datasets.

	Dataset 1	Dataset 2	Dataset 3	‍Dataset 4
‍Type	Sandy loam soil	High porosity quartz sand	Low porosity quartz sand	Porous polycrystalline diamond
Dimensions	512 x 512 x 512	512 x 512 x 512	512 x 512 x 512	‍480 x 480 x 480
Resolution	24 µm	4.5 µm	4.5 µm	‍1 µm
Pore space in voxels	22720090	74766570	57350410	‍8496505
Pore space ratio	17%	57%	42%	‍7.68%
Number of regions	18508	69342	68090	‍68995
Number of balls	191583	582064	478191	‍273190
Adjacencies for regions	61806	307059	280008	192551
Adjacencies for balls	647409	1671656	1950209	619384
Global Connectivity Indicator (regions)	3.34	4.42	4.11	3.52

Data sets 2,3,4 were chosen for testing computationally the model on various pore space structures. Indeed, our carbon turnover scenarios were realistic for data set 1 but somehow artificial (proof for concept) for datasets 2,3,4.

#### 2.4.2 Calibration of diffusion processes in the graph.

In order to use our geometrical representation for the (numerical) simulation of diffusion processes, we calibrate it by comparison with other approaches following the scheme described in section 2.3 (Monga et al. [17]). Here, the goal of the calibration phase is to define the value of the diffusive overall conductance $\propto_{i,j}$.

To carry out this calibration, we introduce a given mass of organic matter (100 µg) into the first two planes at the inlet end of the simulated domain. We stress that, in the case of this simulation, no transformation processes were implemented. We use the molecular diffusion coefficient of DOM in water of 6.73 10−6 cm^2^ s^-1^. After short time period (1.8 hours), we compare the simulation results of the diffusion process through different cross-sections within the system using the two geometrical representations of the pore space: ball-based (MOSAIC) already calibrated using LBM [17], and region-based (approach of the present paper). For the voxel-based approach, the Lattice Boltzmann Method was used to simulate diffusion processes. For the balls- and regions-based approaches, as explained below, graph updating allows to simulate diffusion.

#### 2.4.3 Simulation of the microbial transformation.

To simulate the microbial mineralization of organic matter, the biological parameters that Monga et al. [18] considered for *Arthrobacter sp. 9R* were taken for DOM degradation with 9.6 day^-1^ for the maximum growth rate, 0.001 gC g^-1^ for the constant of half-saturation, 0.2 day^-1^ for the respiration rate, 0.5 day^-1^ for the mortality rate and 0.55 for the portion of microbial biomass that returns to DOM. A rate of 0.001 day^-1^ for the mineralization rate of SOM.

For dataset 1, we put 5.2 10^7^ bacteria (0.18 µg biomass) divided into 1000 spots (each spot corresponding to 5.2 10^4^ bacteria) in the pore space, and a mass of DOM of 0. 2895 mgC in the pore space corresponding to the concentration of 0.13 mgC/g soil [22]. We initially set the masses of FOM and SOM to 0. Due to the transformation processes implemented, the FOM mass sticks to 0 and SOM is created due to micro-organisms mortality. Details of the calculation are provided in reference [22].

For datasets 2,3, and 4, comparison was carried out simply with MOSAIC, involving the network of spherical balls, since the latter has already been compared favorably to the simulations with the LBM method [10]. The biological parameters and the initial masses of micro-organisms and DOM were the same as for dataset 1 up to the resolution change (biomass: dataset 1: *0.18 µg*, dataset 3: *0.001 µg*), and the Dissolved Organic Matter (organic matter: dataset 1: *0. 2895 mgC*, dataset 3: *0.001979 mgC*) was likewise, at first, distributed homogeneously in the pore space. We put two spots of micro-organisms in the two biggest balls, with a radius of 27 and 30 voxels, respectively, considered in the Mosaic ball-based simulations.

We point out that, for our chosen examples as in most cases, the simulation results will be identical for long periods (months, years, centuries…) but not for shorter periods (days, weeks...). Indeed, in the tested scenarios, DOM was not renewed through hydrolysis of FOM (Fresh Organic Matter). After enough time, the whole microbial and organic matter masses will be transformed into carbon dioxide. Also, in the case where the pore space is not completely connected, bits of organic matter can stay in some locations if not accessible by micro-organisms.

#### 2.4.4 Relevance of the spatialization of the dynamics.

Let us now discuss the interest about spatializing precisely the model. Practically the dynamics can be very different depending on the pore space geometric structure and on the initial location of the micro-organisms and the organic matter. Theoretically (see PDE system of section 2.3.3) the biological dynamics is governed by the mesh (graph) describing the pore space (especially the pore connectivity) and by the initial conditions (placement of the materials at starting time). Of course, when dealing with real soils (like in the data of the present paper), its influence is much more important than in the case of reworked soil. An efficient way to evaluate the impact of the soil heterogeneity, is to compare the curves describing the dynamics with spatialization and without spatialization. We simulate the corresponding ODE-based model without spatialization (OD model), which considers only transformation processes without diffusion, applied to the total masses of different compounds. We then compare it to the spatialized (3D) model, where transformation and diffusion processes are applied to the same initial amount of microorganisms distributed using 1000 bacterial clusters of varying sizes. This patchiness, influenced by cell growth mechanisms and environmental constraints, aligns with observations made by Nunan et al. [33]. The DOM mass is homogeneously distributed (i.e., the same concentration in all regions).

## 3 Results

### 3.1 Geometrical modelling of the pore space

Regarding computational cost and memory requirements, the segmentation effort of the curve skeleton based method can be split into:

- Computation of the curve skeleton
- Segmentation of the curve skeleton into simple branches
- Partition of the pore space using the maximal simple branch.

Practically these three steps need less computational effort than the approximation schemes based on geometrical primitives (balls, ellipsoids, generalized cylinders…). The memory requirements of the two approaches are close.

The geometrical modelling of the pore space using the approach based on the curve skeleton (see section 2.2) was performed for each of the four images considered, when performed on a regular midrange laptop computer (AMD Ryzen 5 5500U, 8 Gigabytes RAM). The different computing times for the four data sets were, respectively, 61 seconds (data set 1, sandy loam soil), 124 seconds (data set 2, Fontainebleau quartz high porosity), 101 seconds (data set 3; Fontainebleau quartz low porosity), and 48 seconds (dataset 4, porous polycrystalline diamond). In terms of the associated demand of CPU time, one should note that this geometrical modelling has to be performed only once before simulating processes within the porous materials, including the various biological dynamics scenarios.

It is relatively straightforward to illustrate graphically on the 2D cross sections how the resulting partitioning looks, when each partitioned pore region is depicted with a different color (Fig 1e). However, the connections existing between the different regions are more evident when one zooms-in in 3D inside a portion of the image (Fig 3). This zooming-in highlights the compacity and the coherence of the pore regions.

In Figs 1e, 3, 4 and S2, the colors correspond to the set of voxels attached to one subset forming a region that is the closest voxels to simple branches. In the microbial calculation, every colored subset is represented by its inertia center, its volume, (and the micro-organisms and organic matter masses). The areas of the contact surfaces are attached to the graph edges. We also keep a label image allowing to get back to all voxels forming a region.

We also calculated the values of the Global Connectivity Indicator (CGI) defined by the ratio number of arcs/number of nodes for both geometrical models (balls, regions). For data set 1 we got 18508 regions (pores) and 61806 arcs (adjacencies). The ball network included 191583 balls and 647409 arcs. Therefore the GCI was 3.34 for curve skeleton model and 3.38 for balls model, which are close values. For data set 3 we got 68090 regions (pores) and 280008 arcs (adjacencies). The ball network included 478191 balls and 1950209 arcs. Thus the GCI was 4.11 for curve skeleton model and 4.08 for balls model, which are also close values. The above computation of GCI demonstrated the good concordance of balls model and curve skeleton model regarding the degree of connectivity.

### 3.2 Calibration of diffusion processes

In order to calibrate the diffusion processes taking into account the graph, we use the method described in section 2.4.2. The comparison of the different depth-concentration profiles obtained after adjustment of the conductance with the region-based approach with those resulting from the Mosaic-based approach (itself already compared in the past with the LBM method) suggests that an optimal fit of these different methods is obtained by setting $\propto_{i,j}$ to a constant value, equal to 0.35 (Fig 6). The reason is that when the pore shapes are relatively homogeneous the diffusive overall conductance can be considered as constant. In forthcoming research, we shall propose a way to compute it for each pair of connected regions, which is more precise but also more costly in term of computing time.

### 3.3 Modelling of microbial mineralization including diffusion processes

We use the framework described in section 2.3. As mentioned, the computational cost is roughly proportional to the number of graph nodes which is the number of balls for the ball based method. In most cases, the number of regions provided by the curve skeleton-based method is much lower (ratio of 1/10–1/20).

The computational cost of the diffusion phase is roughly proportional to the number of nodes of the geometrical primitive graph. For data set 1, using the geometrical modelling scheme described by Monga et al. [20], 191583 balls are involved in the description of the pore space. The use of the curve skeleton-based algorithm described here results in the identification of 18508 regions. Thus, the computing time for the diffusion simulation is divided by a ratio of approximately ten. For dataset 3, we obtained 478191 balls and 72691 regions, yielding a ratio of 7. For dataset 4, we obtained 407055 balls and 45386 regions, yielding a ratio of 9. For dataset 2, we obtained 2376261 balls and 259299 regions, yielding a ratio of 9 (see Table 1).

The microbial mineralization of organic matter is described as the second step of a two-step sequence, the first step involving the diffusion of dissolved organic molecules, which is described using an implicit numerical scheme.

For dataset 1, we compare the results obtained by using the skeleton-based pore network graph with the ones obtained under identical conditions in an earlier publication [17] via the use of LBM and MOSAIC (Fig 7). Although there are some small numerical differences between the curves obtained with these different methods, their overall similarity is particularly noteworthy, given the large differences in computing times they require. On the personal computer that we used for the calculations, the computing time (CPU) for the LBM approach was several days; the Mosaic ball-based approach took 20 minutes, and the new method, based on partitioning the pore space with the curve skeleton, yielded its result in one minute.

In the specific case of dataset 2–4 these balls correspond roughly to the volume of the 10 biggest regions when using the curve skeleton-based approach. To be precise, 192 regions are included in the two biggest balls but about 85% of the ten biggest regions are included within the two biggest balls. It is due to the fact that the biggest spheres are positioned in the middle of the pore where the regions ends. Therefore, to enable a comparison between the two different simulation methods, we placed in these regions the same biomass as in the two largest balls in the Mosaic simulations (see Fig 4). We also adjust the diffusion coefficient of carbon (DOM) in water (dataset 1: *100k voxel*^*2*^*/day*, dataset 3: *2500k voxel*^*2*^*/day)*. We keep the same values for the diffusive conductive coefficients (*0.35* for the curve skeleton model, *0.6* for the balls model).

Simulation results for the data set 3 (Fig 8) show that the different curves are close for the two methods, in spite of considerable differences in the time required for computation. The simulations took 1 hour of CPU time with the Mosaic approach, versus merely one minute with the curve skeleton-based model. The results in Fig 8 also demonstrate that the diffusive overall conductance coefficient does not need (at least for these datasets) to be updated when changing datasets. Similar agreements between the two simulation methods were obtained for data sets 2 and 4 (see section 8: “supplementary material”), as well as a comparable 10-fold speeding up of the time needed for completion.

### 3.4 Spatialization relevance: Comparison with ODE model

In order to show the relevance of the dynamics spatialization we use the scheme described in section 2.4.4.

**Fig 9:**
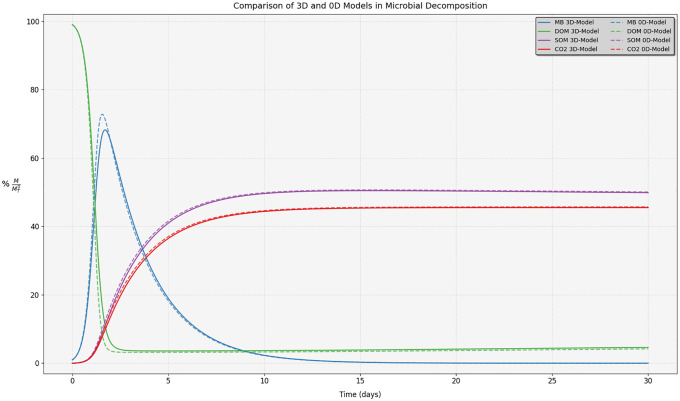
Comparison of 3D and 0D (non-spatialized) models for microbial decomposition. For this specific simulation, we set Vsom = 0, that explains why DOM drops to 0 although MB and SOM are positive.

**Fig 10:**
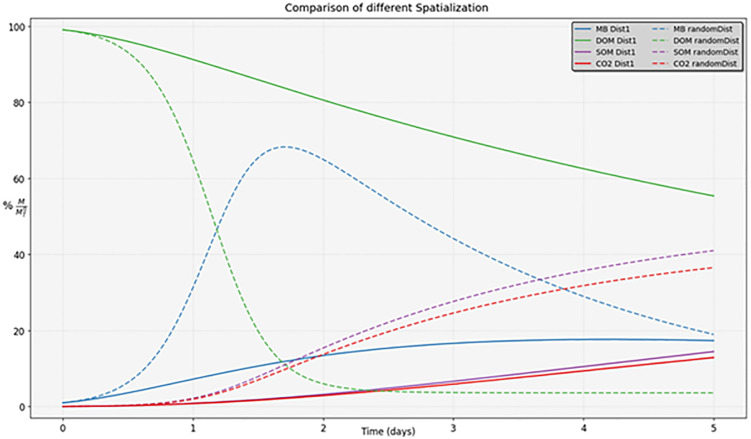
Comparison of different spatializations for the 3D model. The principle of our method consists of segmenting the curve skeleton into simple branches, and afterward attaching to each simple branch a connected set of points. The result is a partition of the pore space, which can be represented by an attributed relational graph. In this graph, each node corresponds to a pore, and each arc to an adjacency between a couple of pores. We show that this geometrical representation of the pore space can be used directly to simulate the microbial mineralization dynamics including diffusion and transformation processes. We assess the soundness of the approach by comparison with other methods that have been developed and used in the past in the same context. A possible drawback of the novel approach, compared with geometrical primitives-based modeling, is that each pore is defined by a set of connected voxels with no explicit geometric properties. On the other hand, the advantages of the pore space modeling based on the curve skeleton are fourfold. It does not impose specific shapes for a pore as do primitive-based modeling methods, involving balls or ellipsoids. It defines an exact piece-wise representation of the pore space, without losing any part of it, since it is based strictly on a partitioning of the pore space into distinct regions. On the other hand, the computations on the basis of the curve skeleton use reduced information attached to the valuated graph (inertia center, region size, contact surface) that is the limitation of this framework. When implementing diffusion processes, it involves an exact computation of the area of the contact surfaces between adjacent regions. Finally, the number of nodes (pores) is much less than the ball network used in a previous work. To maximize the advantages and minimize the drawbacks, it might be possible in the future to implement hybrid geometric modeling algorithms using both skeletonization and geometric primitives.

The comparison shows that the simulation results of the ODE model differ significantly from those of the 3D model, at least when the localization of micro-organisms is not random (see Figs 9, 10). This is expected, as micro-organisms in the ODE model have complete access to the total DOM, leading to exponential growth. Once the DOM is fully consumed, the microorganisms begin to die and are subsequently transformed into DOM and SOM. We notice that the low value of the decomposition rate of SOM set to 0.001 day-1, explains why DOM drops to 0 although MB and SOM are positive. The biomass recycling into DOM and the low transformation rate of SOM into DOM are almost entirely taken up and respired by the biomass. We ran the ODE simulation of Fig 9 over much longer periods of time and after about 30 years, DOM, MB and SOM converge to zero while almost all the initial masses were transformed into CO_2_. Interestingly, we observed that the scenario with a random localization of microorganisms produced results close to those of the ODE model (without spatialization).

In our second experiment, we conducted two simulations using the 3D region-based model, each with different spatial distributions of the same initial amount of microorganisms, the first distribution is the same as the first experiment, and in the second distribution, we choose random spots of microorganisms. The results demonstrate that the placement of microorganisms within the pore space significantly influences the system behavior. The results are shown in Figs 9, 10.

## 4 - Discussion

This work brings the following outcomes:

- Curved skeleton, already used to model complex shapes within various contexts, can also be employed for pore space geometric representation in soil science.
- Such curve skeleton based pore space representation allows to speed up hugely the computational cost of microbial decomposition simulation.
- Biological dynamic simulation schemes using three different geometric representations of pore space (voxels, balls, regions) provide similar results but with very different computing times (up to 1/10000 for voxels versus regions).

Its main limitations are:

- Calibration of the diffusion coefficient (diffusive conductive coefficient) is needed when switching from one geometric representation to another one
- Time steps for Euler numerical schemes for both transformation and diffusion processes are defined on an ad hoc basis. Practically, enough small time steps are chosen.
- Preprocessing steps on grey level CT images can differently remove noise and therefore introduce slight differences in the resulted segmented pore space that can be large enough to lead to different outputs of the physical models [11].

The possible and future scenarios and perspectives are:

- Complementary developments could be carried out to avoid the diffusion coefficient calibration. Indeed the diffusion is implemented by computing the flow (first Fick law) between two adjacent primitives using the same diffusion coefficient for any pair of adjacent pores. Therefore the calculation of the diffusive conductive coefficients depending on the specific pore shapes could both escape any calibration and also improve the precision of the simulation.
- The determination of optimal (maximal) time steps in Euler numerical schemes for both transformation and diffusion processes could also be explored. Practically we took time steps such that the result is stable when decreasing it.
- Pore space changes along the dynamic could be taken into account during the simulation process
- The stability of our biological model when using different segmented image outputs (resulting from different preprocessing steps) could be investigated and quantified.
- Machine Learning (ML) techniques (Deep Learning, Conventional Neural Network…), instead of computational geometry, could provide pore space partition. Within this context, the main issue would be to get enough training data (massive data) for the ML system. Practically, as far as we know, no available data bases provides enough data yet.

Compared to other schemes using piece wise approximation by geometrical primitives (balls, ellipsoids, generalized cylinders…), this method avoids approximation errors because defining an exact partition of the pore space, without imposing a priori shapes to the pores. In particular, the borders between the regions are straightforward defined. Also the number of graph nodes (corresponding to regions) is less than when using primitives, yielding a more compact representation.

In this work, we assume implicitly that the pore space partition, computed from the curve skeleton, fits reasonably with micro-organisms habitats. We consider the regions as “biological units”. Practically, we do not obtain very large regions due to the generally tormented shapes of pore space. For data set 1 (image resolution: 24µm x 24µm x 24µm), the maximum region size is 15521 voxels (round 0.27 mm^3^) and the median value 1180 voxels (round 0.016 mm^3^). For dataset 3 (image resolution: 4.5µm x 4.5µm) the maximum region size is 20466 voxels (round 0.002 mm^3^) and the median value 484 voxels (round 0.00004 mm^3^).

## 5 Conclusion

The key result of the present article is the demonstration that the use of the curve skeleton to partition the pore space in 3D micro CT (Computed Tomography) images of soil samples, followed by the use of this partition for the simulation of the microbial mineralization of soil organic matter, leads to considerably shorter computing times. As far as we are aware, this use of the curve skeleton to represent complex volume shapes is the first in the context of soils. The method has been used in other disciplines in the past, like medicine, material sciences, chemical engineering, and in the analysis of porous media (e.g., carbonate rocks) but never to speed up the otherwise extremely lengthy simulations needed to describe the fate of organic matter in soils subjected to environmental change.

## Supporting information

S1 Fig Top left: 2D gray scale image of the sand high porosity (dataset 2). Top right: Corresponding binary image. Bottom left perspective view of a part of the pore space. Top right: perspective view of the corresponding curve skeleton.
(TIF)

S2 Fig Corresponding segmented regions of the sand high porosity (data set 2) and the ball network within the same part of the whole 3D image. (TIF)

S3 Fig Comparison of the simulations of microbial mineralization for dataset 2 (sand high porosity) using the ball based MOSAIC program and the novel approach introduced here, based on the partitioning of the pore voxels on the basis of the curve skeleton. The simulations extended over 5 days and were carried out in the “curve skeleton” method with a diffusive conductance coefficient equal to 0.35 for the curve skeleton method and 0.6 for the balls method. The initial masses (micro-organisms, dissolved organic matter) and also the diffusion coefficient were adjusted according to the image resolution. X-axis and Y-axis represents respectively time expressed in hours and the masses expressed in percentage of the total initial masses. Solid line curves and dotted line curves correspond respectively to the curve skeleton model and to the balls model. Dark blue curves, green curves, red curves, light blue curves correspond respectively to microorganisms, dissolved organic matter (DOM), carbon dioxide (CO_2_), soil organic matter (SOM). Same as for dataset 1 the microbial degradation model take into account DOM and SOM but not FOM.
(TIF)

S4 Fig Top left: 2D gray scale image of the carbonate rock dataset. Top right: Corresponding binary image. Bottom left: perspective view of a part of the pore space. Bottom right: perspective view of the corresponding curve skeleton.
(TIF)

S5 Fig Comparison of simulations of microbial mineralization for dataset 4 (carbonate rock) using the ball based MOSAIC program and the novel approach introduced here, based on the partitioning of the pore voxels on the basis of the curve skeleton. The simulations extended over 5 days and were carried out in the “curve skeleton” method with a diffusive conductance coefficient equal to 0.35 for the curve skeleton method and 0.6 for the balls method. The initial masses (micro-organisms, dissolved organic matter) and also the diffusion coefficient were adjusted according to the image resolution. X-axis and Y-axis represents respectively time expressed in hours and the masses expressed in percentage of the total initial masses. Solid line curves and dotted line curves correspond respectively to the curve skeleton model and to the balls model. Dark blue curves, green curves, red curves, light blue curves correspond respectively to microorganisms, dissolved organic matter (DOM), carbon dioxide (CO_2_), soil organic matter (SOM). Same as for dataset 1 the microbial degradation model take into account DOM and SOM but not FOM.
(TIF)

S6 FigConvergence of the simulation method (transformation + diffusion).We present the biological dynamics curves obtained for different time steps for dataset 1. Up figure: kinetic for DOM, CO_2_, SOM, Biomass (left to right and up to bottom) when using different discretization time steps δt (time steps are expressed in seconds).(TIF)

S7 Fig Convergence of the diffusion method (dataset 1). The meaning of the curves is the same as in Fig 7. The X-axis displays the number of planes within the image (512 planes in total), whereas the Y-axis displays the total mass of organic matter within each plane. At the start, 100 µg of carbon were introduced within the first two planes. The total simulation time was 1.783 hours. We implemented the diffusion process using Euler backward scheme (implicit scheme) with different decreasing discretization time steps in order to show the convergence in time. We display the curves showing the total amount of matter for each planar cross section (z-planes) after the diffusion process. Upper left: discretization time step (implicit Euler scheme) was set to 1.783h; the red curve corresponds to the ball network and the blue one to the curve skeleton based network. Up right: same as up left but where the discretization time step was set to 30s. Bottom left: curves obtained using the curve skeleton based network for decreasing time steps; we see the convergence of the process. time step = 1.783h, 0.90h, 30s…0.13s. The convergence is approximately reached when time step is set to 30s. Bottom right: same as bottom left but with zooming in some areas.
(TIF)
