# Supplementary figures and images for: Geometric modelling of 3D pore space using curve skeleton: Application to computational microbiology of soil organic matter mineralization
Source: PLoS One. 2025 Nov 7;20(11):e0331031. doi: 10.1371/journal.pone.0331031 (PMC12594357; doi:10.1371/journal.pone.0331031)

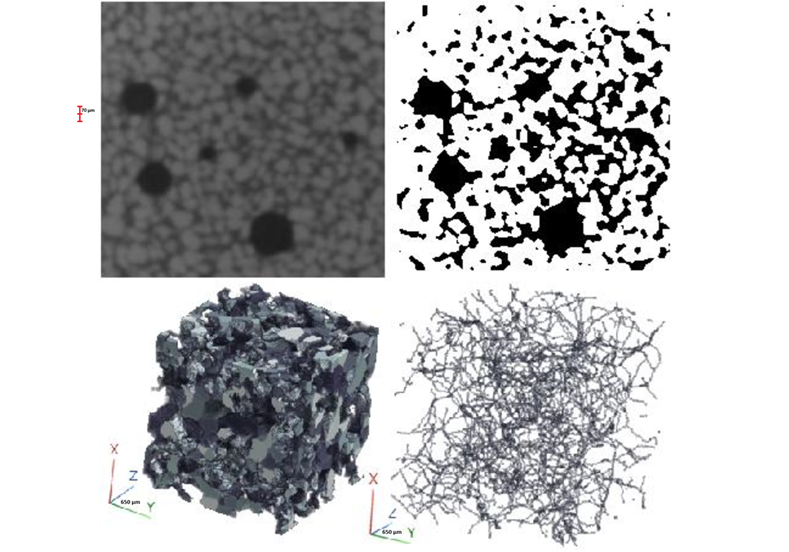

Supplement: S1 Fig — Top right: Corresponding binary image. Bottom left perspective view of a part of the pore space. Top right: perspective view of the corresponding curve skeleton. (TIF) [file pone.0331031.s001.tif]

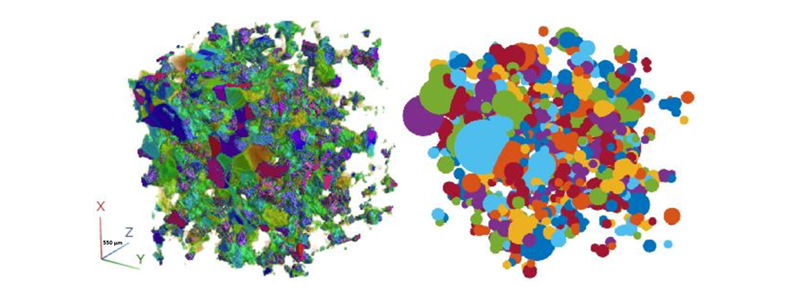

Supplement: S2 Fig — (TIF) [file pone.0331031.s002.tif]

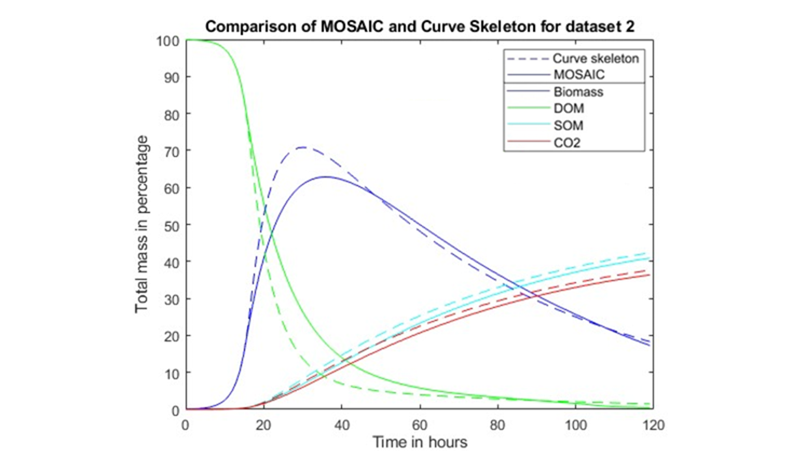

Supplement: S3 Fig — The simulations extended over 5 days and were carried out in the “curve skeleton” method with a diffusive conductance coefficient equal to 0.35 for the curve skeleton method and 0.6 for the balls method. The initial masses (micro-organisms, dissolved organic matter) and also the diffusion coefficient were adjusted according to the image resolution. X-axis and Y-axis represents respectively time expressed in hours and the masses expressed in percentage of the total initial masses. Solid line curves and dotted line curves correspond respectively to the curve skeleton model and to the balls model. Dark blue curves, green curves, red curves, light blue curves correspond respectively to microorganisms, dissolved organic matter (DOM), carbon dioxide (CO2), soil organic matter (SOM). Same as for dataset 1 the microbial degradation model take into account DOM and SOM but not FOM. (TIF) [file pone.0331031.s003.tif]

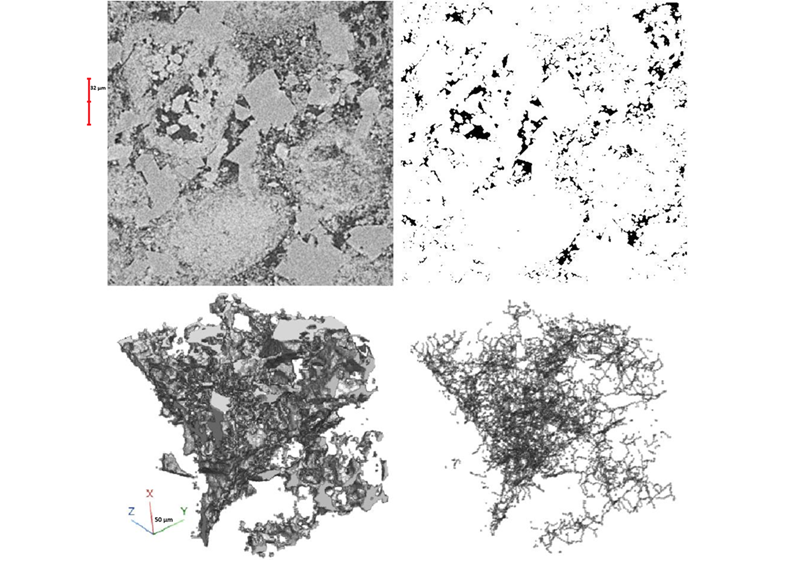

Supplement: S4 Fig — Top right: Corresponding binary image. Bottom left: perspective view of a part of the pore space. Bottom right: perspective view of the corresponding curve skeleton. (TIF) [file pone.0331031.s004.tif]

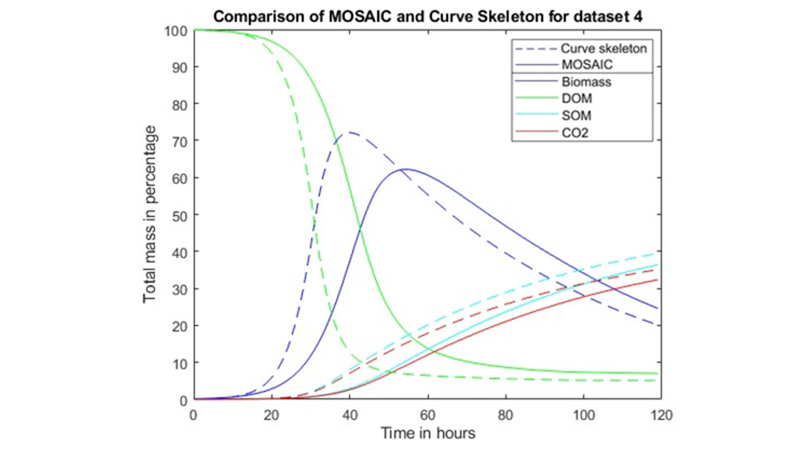

Supplement: S5 Fig — The simulations extended over 5 days and were carried out in the “curve skeleton” method with a diffusive conductance coefficient equal to 0.35 for the curve skeleton method and 0.6 for the balls method. The initial masses (micro-organisms, dissolved organic matter) and also the diffusion coefficient were adjusted according to the image resolution. X-axis and Y-axis represents respectively time expressed in hours and the masses expressed in percentage of the total initial masses. Solid line curves and dotted line curves correspond respectively to the curve skeleton model and to the balls model. Dark blue curves, green curves, red curves, light blue curves correspond respectively to microorganisms, dissolved organic matter (DOM), carbon dioxide (CO2), soil organic matter (SOM). Same as for dataset 1 the microbial degradation model take into account DOM and SOM but not FOM. (TIF) [file pone.0331031.s005.tif]

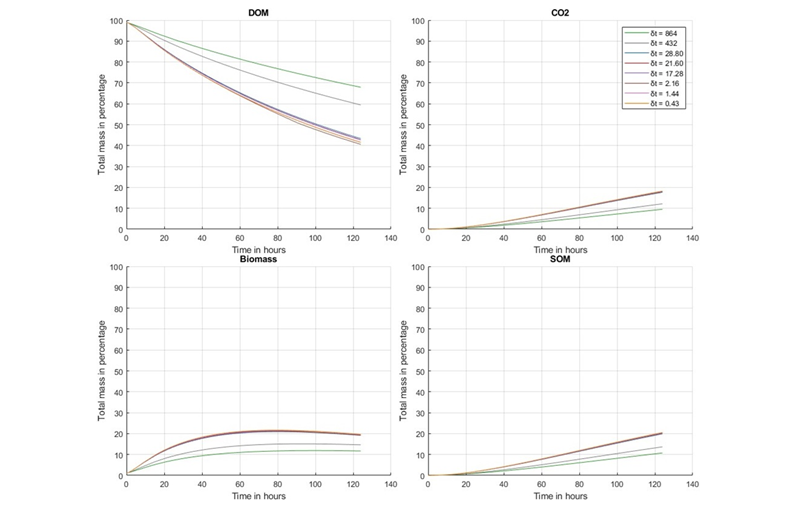

Supplement: S6 Fig — We present the biological dynamics curves obtained for different time steps for dataset 1. Up figure: kinetic for DOM, CO2, SOM, Biomass (left to right and up to bottom) when using different discretization time steps δt (time steps are expressed in seconds). (TIF) [file pone.0331031.s006.tif]

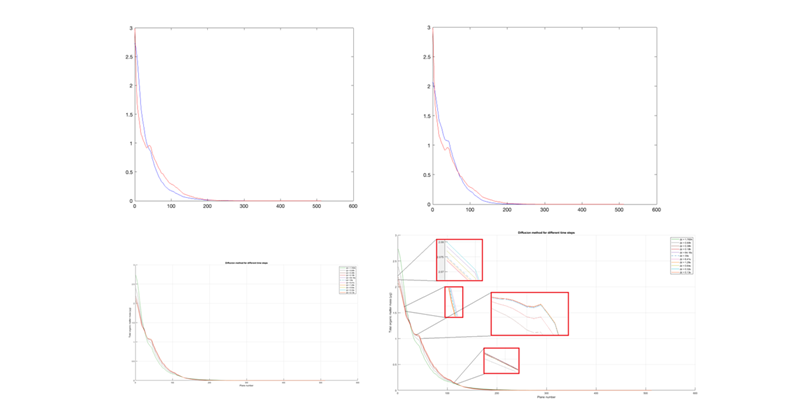

Supplement: S7 Fig — The meaning of the curves is the same as in Fig 7. The X-axis displays the number of planes within the image (512 planes in total), whereas the Y-axis displays the total mass of organic matter within each plane. At the start, 100 µg of carbon were introduced within the first two planes. The total simulation time was 1.783 hours. We implemented the diffusion process using Euler backward scheme (implicit scheme) with different decreasing discretization time steps in order to show the convergence in time. We display the curves showing the total amount of matter for each planar cross section (z-planes) after the diffusion process. Upper left: discretization time step (implicit Euler scheme) was set to 1.783h; the red curve corresponds to the ball network and the blue one to the curve skeleton based network. Up right: same as up left but where the discretization time step was set to 30s. Bottom left: curves obtained using the curve skeleton based network for decreasing time steps; we see the convergence of the process. time step = 1.783h, 0.90h, 30s…0.13s. The convergence is approximately reached when time step is set to 30s. Bottom right: same as bottom left but with zooming in some areas. (TIF) [file pone.0331031.s007.tif]
